# Creating a healthcare variant CYNEFIN framework to improve leadership and urgent decision-making in times of crisis

**DOI:** 10.1108/LHS-03-2021-0013

**Published:** 2021-08-16

**Authors:** Paul James Lane, Robyn Clay-Williams, Andrew Johnson, Vidula Garde, Leah Barrett-Beck

**Affiliations:** Townsville Hospital and Health Service, Queensland Health, Townsville, Australia; Human Factors and Resilience Research, Australian Institute of Health Innovation, Macquarie University, Sydney, Australia; Townsville Hospital and Health Service, Queensland Health, Townsville, Australia; Metro North Health, Queensland Health, Brisbane, Australia

**Keywords:** Resilience, Crisis management, Leadership, CYNEFIN, Complexity, Decision-making

## Abstract

**Purpose:**

The complex and occasionally chaotic nature of health care has been previously described in the literature, as has the broadening recognition that different management approaches are required for different types of problems rather than a “one size fits all” approach. The CYNEFIN framework from Snowden outlines a consistent cognitive approach that offers the leader and leadership team an ability to urgently apply the correct actions to a given situation. This paper proposes a variant CYNEFIN approach for healthcare.

**Design/methodology/approach:**

Consistent and accurate decision-making within health care is the hallmark of an effective and pragmatic leader and leadership team. An awareness of how one’s cognitive biases and heuristics may adversely impact on this cognitive process is paramount, as is an understanding of the calibration between fast and slow thinking.

**Findings:**

The authors propose a variant CYNEFIN approach for health care of “act-probe-sense-respond” to resolve complex and time-critical emergency scenarios, using the differing contexts of a cardiac arrest and an evolving crisis management problem as examples. The variant serves as a pragmatic sense-making framework for the health-care leader and leadership team that can be adopted for many time-critical crisis situations.

**Originality/value:**

The variant serves as a pragmatic sense-making framework for the health-care leader that can be adopted for many crisis situations.

## Introduction

Cognitive errors account for a large number of clinical incidents leading to patient harm and death (Croskerry, 2003; Royce *et al.*, 2019; Wilson *et al.*, 1995). It has been recognized that cognitive biases have a significant adverse impact on decision-making performance in health care (Croskerry, 2013; Saposnik *et al.*, 2016). Kahneman describes System 1 and System 2 thinking as an approach used by the human brain to enable problem-solving (Kahneman, 2011). Experts seemingly often intuit what is happening and a quick response is generated, predicated on the selection of the appropriate preformed script of thoughts and actions based upon previous experience, termed System 1 thinking. They often use “rules of thumb” (known as heuristics), which are pragmatic, simple approaches to complex scenarios that have been found often, but not always, to be helpful (Marewski and Gigerenzer, 2012). Novice clinician leaders and health-care managers, however, should rely upon System 2 thinking: a slow, first-principles approach, capable of only handling 5–7 inputs of data and associated with the significant cognitive burden. A novel problem frequently requires integration of both approaches to reach the best decision, for a fatal error for the expert, especially under time pressure, is to choose a preformed but incorrect script, and thus pursue a positive outcome based on past experiences rather than the actual facts of the problem at hand (Croskerry, 2013; Croskerry *et al.*, 2013; Graber *et al.*, 2012).

While an understanding of these cognitive processes is extremely helpful for an individual or an organization that is dealing with chaos and ambiguity due to crisis, a cognitive framework approach enhances collaboration and communication across a team, thus delivering highly reliable performance (Cooke *et al.*, 2012).

## The CYNEFIN framework and health-care complexity

**Figure 1.**
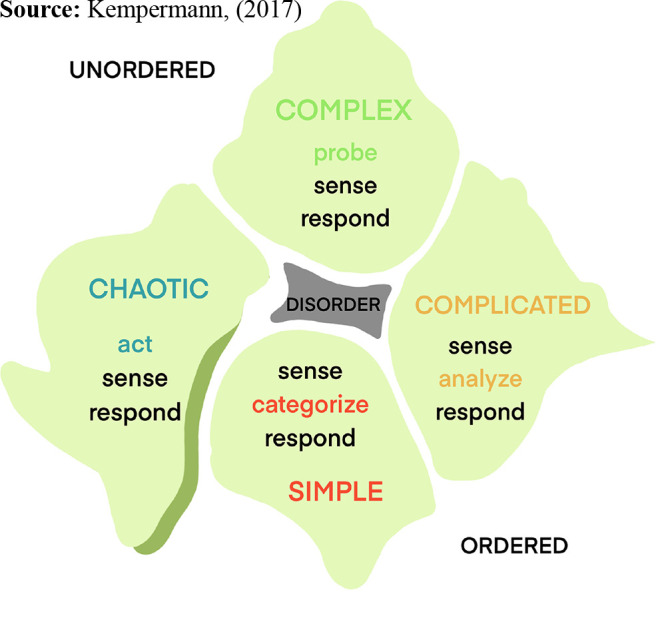
CYNEFIN sense-making framework

Snowden’s CYNEFIN sense-making framework, illustrated in Figure 1, has been shown to have a degree of applicability to health care (Baker *et al.*, 2006; Burman and Aphane, 2016; Kempermann, 2017; Mark and Snowden, 2006; Snowden, 2000; Snowden and Boone, 2007; Van Beurden *et al.*, 2013). Recent applications have been proposed to assist with managing the COVID-19 pandemic (Rubin and de Vries, 2020; Snowden and Rancatti, 2021). With linear systems, where clear cause and effect relationships associated with malfunction can be determined, reliability can be achieved through policies, procedures and standardization. Even in complicated problems, analyzes can help establish linearity. Those situations with a high degree of complexity and unpredictability with increasing chaotic potential present an even greater challenge where clear cause and effect relationships are difficult or indeed impossible to establish. These chaotic problems require a resilient approach, based upon first principles, with initial actions to create some order and inhibit accident propagation (Baker *et al.*, 2006; Johnson *et al.*, 2019; Veazie *et al.*, 2019). For the novice leader perhaps the most significant domain of concern within the CYNEFIN framework is disorder, where a leader believes they are dealing with a simple or obvious problem, when indeed a complex or chaotic problem-solving approach is required. Unfortunately, such mismatching of the problem and action required facilitates sub-optimal performance where recovery is difficult to achieve (Snowden, 2010; Snowden and Boone, 2007).

Leadership in health care encompasses challenges across all five domains of the CYNEFIN framework, however, the more experienced leader will no doubt be called upon to resolve the more complex and chaotic situations. Many current leaders rely upon individual experience, preferred change methodologies and personal characteristics such as charisma to achieve success. Unfortunately, for many leaders who are faced with a new or novel crisis, “black holes” in knowledge can emerge and critical mistakes are made. Mitigating this scenario is a sound teamwork-based approach such as Crew Resource Management, where teams can assist the leader with the problem-solving through a flattened hierarchy and potentially compensate for leadership weakness if given permission to do so (Kosnik, 2002; McConaughey, 2008).

Further, some errors in judgment highlight the limits of the current approach to the way problem-solving is taught in medicine. The traditional clinical approach of history, examination and investigations with subsequent generation of differential diagnosis leading to treatment based upon evidence in a linear fashion predominates, rather than the actual real-world environment (Talley and O’Connor, 2014). Here, within the modern health-care industry that is recognized for volatility, uncertainty, chaos and ambiguity (VUCA), there is frequently the need for immediate treatment that is often reliant on a “first principles” approach or “gut instincts” operating with incomplete information in an unstable environment to render the situation safe (Billiones, 2019). These initial action plans exist in parallel with the ongoing thorough patient or management problem assessment (Early Trauma Care: Primary Survey, 2021; Georgiou and Lockey, 2010).

Importantly, health care has been previously described as having features of complex adaptive systems (Braithwaite *et al.*, 2013, 2021). These systems are characterized by: a lack of hierarchical order, the following of simple rules, the dependence upon cohesion between the various agents within it, the display of emergent behavior, a predisposition to the “butterfly effect,” and the exhibiting of “Edge of Chaos” phenomenon (Rouse, 2008). The “Edge of Chaos” is a natural, semi-structured state between order and chaos. In the context of health care, some have described it as “freefall” with a sense of the loss of control (Wears *et al.*, 2006). If left unmitigated, this potential crisis may plunge the system further toward chaos and an ensuing accident or serious clinical incident. Paradoxically, if such loss of order is contained by boundaries and well-managed with a proper concern for safety, the edge of chaos also allows for system innovation and adaptation (Forrest, 1999; Pozzo *et al.*, 2015; Snowden and Rancatti, 2021). Health-care leaders need to recognize the positive and negative aspects of operating in proximity to the safety boundary, knowing that moments of chaos are inherent to delivering patient care in hospitals and health-care organizations. Further, these leaders may benefit from a modified CYNEFIN approach, as described below, to help solve time-critical crisis scenarios.

## Act-probe-sense-respond: a CYNEFIN variant

**Figure 2.**
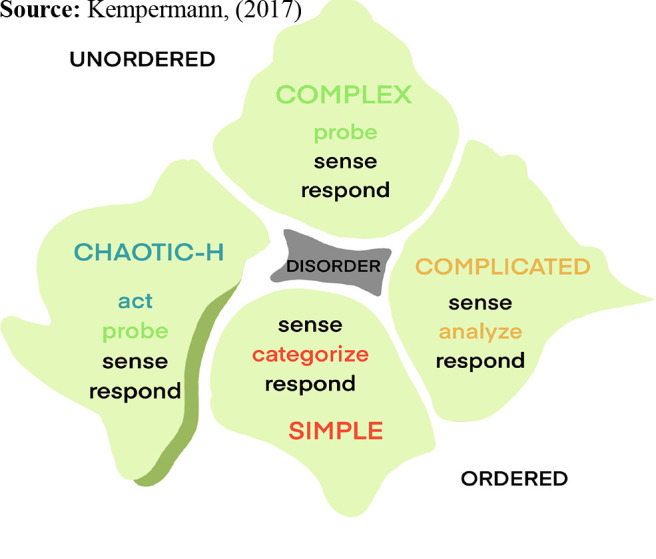
CYNEFIN health-care variant sense-making framework

Snowden advocates a Probe-Sense-Respond approach when dealing with complexity and an Act-Sense-Respond process in dealing with chaotic situations such as a crisis. By aligning these two unordered approaches to create a health-care variant CYNEFIN approach of act-probe-sense-respond (Figure 2), we propose the health-care leader and their team is able to deal immediately with the chaos and recognize and manage the co-existent complexity of the underpinning problems. There must be an action initiated by the leader to create some degree of order to halt the propagation of the chaos, rendering safe a potentially dangerous situation with the least invasive intervention possible. Inhibiting error progression promotes resilient performance and the creation of satisfactory order allows other team members to respond, even if they themselves are confused by the complexity of the overall scenario (Baker *et al.*, 2006). Edmonson describes this leadership trait in complex and uncertain situations as “teaming,” enabling team performance while learning and planning (Edmondson, 2012). Scharmer and Senger similarly describe “prototyping,” a process by teams where imperfect concepts can be presented to facilitate fast-cycle learning and adaptation (Scharmer and Senge, 2016). The probing for further information and iterative sense-making by the leadership team allows for more definitive solution responses to be enacted, moving the system away from the edge of chaos. We illustrate our proposed approach through two scenarios.

## The deteriorating patient

Often in chaotic situations in acute health care, such as an acutely deteriorating patient with failing vital signs, the clinical leader must act to create order, even the absence of all the facts: IV fluid can be ordered even if the cause of the tachycardia and hypotension are yet to be elucidated. Simultaneously, in parallel, there are attempts to probe for clinical signs and further background information about the patient. Dependent on the time-scale used, the clinician may be acting, then probing in a series; for example, stopping the bleeding, then finding the source of the bleeding or in other cases, it may be observed that the resuscitation (act) and assessment (probe) are occurring in parallel. Once these efforts are made to stabilize the patient, sense-making can occur with a more formal, informed response to follow.

It is interesting to examine the potential problems that are addressed in the chaos of a cardiac arrest by the basic life support paradigm of DRSABCD: danger, response, shout for help, airway, breathing, cardiopulmonary resuscitation and defibrillation. Recognizing this pathway has evolved over time and represents best practice, upon reflection it also bears significant similarities to what is proposed by the CYNEFIN hybrid model. There is an initial action; look for danger, followed by a probe; check for responsive, a simple sense-making of yes/no, followed by the response of sending/shouting for help. This is followed by action: open the airway and a probe: Is the patient breathing normally? In a patient who is not moving, not responding and not breathing normally, cardioplumonary resuscitation (CPR) can be commenced; sense-making and then a definitive response. Further, the pads of an automated external defibrillator should be applied (action), with a cardiac rhythm obtained (probe) and then interpretation by the device (sense-making), followed by a response; to push the flashing red button and defibrillate the patient if indicated. In essence, what is described is three cycles of act-probe-sense-respond, with the establishment of a degree of order: a patient with CPR and defibrillation in progress. It is notable that the initiating actions attempt to inhibit error propagation, such as the first responder also being electrocuted.

## A management crisis – the Friday afternoon special

Management crises for the health-care leader tend to appear in the dying hours of the working week when energy and resources are dwindling and the senior supervisory cover is reduced. This is when the pathology department of your busy general hospital calls to let you know that a clinical staff member has just been confirmed with measles and that they worked with adult and pediatric patients in the busy emergency department while in the infectious period. When such crises develop you are generally not actually on call and have made other plans for the weekend, but as the leader, it falls to you to manage the immediate disaster management response for your hospital.

The first step is to “Act” to render the situation safe with the least intrusive means possible. Initial actions predicated by the context of the crisis should include: identify the team you need for the immediate response, then changing the weekend plans for yourself and the team, conducting a triage meeting as soon as possible with the aim of identifying particularly vulnerable patients quickly and informing the hospital chief executive officer (CEO) of the issue and immediate plans. Further, the infected staff member requires care and support while isolating. The outbreak management team would include senior staff from emergency department, obstetrics, pediatrics, infectious diseases, public health, media relations unit (MRU) and the facility Director of Nursing. Through the professional conduct of chaired triage meetings by the leader, this group would coordinate the required “Probing” for relevant information, for example, gathering information about the high-risk patients seen by the clinician, and attempt to establish the severity of the risk and assign proportionate responses. By flattening the hierarchy and encouraging all team members to contribute, a health-care leader would enable a sensible iterative plan to emerge through “Sense-making” by the group over multiple meetings as information becomes available. Cohesive teams who are experienced with such crises will have the confidence to ask “what are we missing?” and “are we addressing the worst-case scenario?” Next, with agreement from the CEO, formal responses (“Respond”) can be generated for public health actions, media management and providing updates to the government. Responses should also address concerned staff that will require regular updates and support through a stressful time. Credible media responses and management of social media platforms by the MRU will be paramount.

## Conclusion

Successful leadership in health care is characterized by traits that enable high performance at the “edge of chaos,” or indeed deeply within it. Our proposed modification of the CYNEFIN framework to blend the content of the complex and chaos domains offers the health-care leader a sound decision-making framework for problem-solving in whichever context may apply. Further, by realizing the innate complexity and instability of health-care as a VUCA industry with a propensity to “edge of chaos” behavior, the leader can default to the chaos domain and apply the variant approach of act-probe-sense-respond to render the situation safe with the least energy necessary and initiate actions to deal with the ongoing complexity. Importantly, this prevents the CYNEFIN domain of disorder where the complacent leader unknowingly over-simplifies the problem at hand, and thus applies the incorrect framework for the problem-solving which potentiates error progression and plunges the situation into deepening chaos. By calibrating the various modes of thought, the CYNEFIN variant supports the delivery of resilient, safe and high-quality care by promoting optimal actions and decision-making in chaotic situations frequently encountered in health care. It provides the leader with an elegant but simple framework that mitigates against excessive System 1 biases while giving speed, dynamism and fluidity that may be lost in typical System 2 processing. We acknowledge that further research is required to evaluate the effectiveness of the proposed approach and its validity across the plethora of health-care domains.
